# Computing relaxations for the three-dimensional stable matching problem with cyclic preferences

**DOI:** 10.1007/s10601-023-09346-3

**Published:** 2023-06-03

**Authors:** Ágnes Cseh, Guillaume Escamocher, Luis Quesada

**Affiliations:** 1 Institute of Economics, Centre for Economic and Regional Studies, Budapest, Hungary; 2 Department of Mathematics, University of Bayreuth, Bayreuth, Germany; 3 Insight Centre for Data Analytics, Cork, Ireland; 4 School of Computer Science and Information Technology, University College Cork, Cork, Ireland

**Keywords:** Three-dimensional stable matching with cyclic preferences, 3dsm-cyc, Constraint Programming, Relaxation, Almost stable matching

## Abstract

Constraint programming has proven to be a successful framework for determining whether a given instance of the three-dimensional stable matching problem with cyclic preferences (3dsm-cyc) admits a solution. If such an instance is satisfiable, constraint models can even compute its optimal solution for several different objective functions. On the other hand, the only existing output for unsatisfiable 3dsm-cyc instances is a simple declaration of impossibility. In this paper, we explore four ways to adapt constraint models designed for 3dsm-cyc to the maximum relaxation version of the problem, that is, the computation of the smallest part of an instance whose modification leads to satisfiability. We also extend our models to support the presence of costs on elements in the instance, and to return the relaxation with lowest total cost for each of the four types of relaxation. Empirical results reveal that our relaxation models are efficient, as in most cases, they show little overhead compared to the satisfaction version.

## Introduction

Defined on three instead of two agent sets, the 3-dimensional stable matching problem [39] is a natural generalisation of the well-known stable marriage problem [29]. Its most studied variant is the *3-dimensional stable matching problem with cyclic preferences* (3dsm-cyc) [47], in which agents from the first set only have preferences over agents from the second set, agents from the second set only have preferences over agents from the third set, and finally, agents from the third set only have preferences over agents from the first set.

A matching is a set of triples such that each triple contains one agent from each agent set and each agent appears in at most one triple. A *weakly stable matching* does not admit a blocking triple such that *all three* agents would improve, while according to *strong stability*, a triple already blocks if *at least one* of its agents improves, and the others in the triple remain equally satisfied.

Constraint programming approaches allow one to identify instances that do *not* admit a weakly or strongly stable matching—these will be in the focus of our investigation. For such an instance, how to construct a matching that is blocked by only a few triples? Alternatively, which matching minimises the number of justifiably disappointed agents who appear in a blocking triple? A somewhat more sophisticated approach is to assume that a central authority is able to compensate blocking triples or even single agents appearing in blocking triples. If such a compensation has been allocated, then the triple or agent withdraws their claim to form a more advantageous coalition. How to find a matching with the lowest compensation needed to eliminate all blocking triples?

In order to facilitate a general framework, we associate a cost with each agent. The goal is then to minimise the total cost of triples or agents who block the matching, or have to be compensated in order to withdraw from blocking.

### Literature review

We first restrict our attention to related work in the 2-dimensional and non-bipartite stable matching settings. We mention two already established relaxations of stability and also elaborate on problem variants with costs. Then we turn to the 3-dimensional setting, review related work on 3dsm-cyc, and finally discuss constraint models.

#### Relaxing stability

Various stable matching problems need not admit a stable solution. The relaxation of stability by definition necessarily involves the occurrence of blocking pairs. In the literature, two main relaxations have been defined.

The number of blocking pairs is a characteristic property of every matching. A natural goal is to find a matching with the lowest number of blocking pairs; such a matching is called *almost stable*. This approach has a broad literature: almost stable matchings have been investigated in bipartite [9, 32, 34, 38] and non-bipartite stable matching instances [1, 8, 15, 20], but not in the 3-dimensional setting yet.

Agents who appear in blocking pairs in a solution are called blocking agents. Besides minimising the number of blocking pairs, another intuitive objective is to minimise the number of blocking agents [54]. The complexity of minimising the number of blocking agents in a non-bipartite stable matching instance is an open problem that was posed in the seminal book of Manlove [43]. Similar, but slightly more complicated instability measures can be found in the paper of Eriksson and Häggström [24].

#### Costs and preference negotiation in stable matching problems

Arguably the most natural extension of various matching problems is to consider graphs with edge or vertex costs. For bipartite instances with edge costs, finding a minimum-cost stable matching can be done in polynomial time [26, 27, 33, 36]. The same problem for non-bipartite graphs is NP-hard, but 2-approximable under certain monotonicity constraints using LP methods [58, 59].

Vertex costs play a role in stable matching problems if the agents are part of some type of instance manipulation. In their theoretical study, Boehmer et al. [12] allow agents to reshuffle their preference list. College admission is possibly the most widespread application of stability. Surveys report that bribes have been performed in college admission systems in China, Bulgaria, Moldova, and Serbia [35, 42]. However, preference list manipulation, potentially done by assigning money to the affected agents, does not imply an illegal action. The internal assignment process of humanitarian organisations [6, 53, 57] aims at stability in the first place, but it also routinely features salary premium negotiations for staff members sent to a less desirable location.

#### 3dsm-cyc

Several applications areas have been modeled by extended 3dsm-cyc instances. Cui and Jia [22] modeled three-sided networking services, such as frameworks connecting users, data sources, and servers. In their setting, users have identical preferences over data sources, data sources have preferences over servers based on the transferred data, and servers have preferences over users. Building upon this work, Panchal and Sharma [49] provided a distributed algorithm that finds a stable solution. Raveendran et al. [52] tested resource allocation in Network Function Virtualisation. They demonstrated the superior performance of the proposed cyclic stable matching framework in terms of data rates and user satisfaction, compared to a centralised random allocation approach.

A recent real application was described by Bloch et al. [11] who analysed the Paris public housing market. In their work, the first agent set consists of various housing institutions such as the Ministry of Housing, the second agent set is the set of households looking for an apartment, and finally, the third agent set contains the social housing apartments that are to be assigned to these households. Institutions have preferences over household-apartment pairs, and households rank apartments in their order of preference. Cseh and Peters [21] studied a restricted variant where the institutions have preferences directly over the households, no matter which apartment they are matched to.

Maximum relaxations in these applications correspond to the smallest number or cost of users, data sources, servers, households, or housing agencies, who need to be compensated for being part of a blocking triple.

As for the complexity of 3dsm-cyc, Biró and McDermid [10] showed that deciding whether a weakly stable matching exists is NP-complete if preference lists are allowed to be incomplete (and each agent can only be matched to agents on their incomplete preference list), and that the same complexity result holds for strong stability even with complete lists. However, the combination of complete lists and weak stability proved to be extremely challenging to solve. After a series of papers [13, 25, 50] proving that small 3dsm-cyc instances always admit a weakly stable matching, Lam and Plaxton [41] recently showed NP-hardness for instances with at least 90 agents per agent set—this is also the size of the smallest known no-instance.

#### CP models for 3dsm-cyc

Several constraint models have been developed for the bipartite stable matching problem and its many-to-one variant [31, 44, 48, 55, 60, 61]. We build upon the recent work of Cseh et al. [17], who introduced five constraint models for 3dsm-cyc. Besides capturing both weak and strong stability, they translated three fairness notions into 3-dimensional matchings.

### Our contribution

In this paper we study four types of relaxation to 3dsm-cyc, based on two established and two new relaxation principles. For each of these types we propose CP approaches that are built on top of the best two approaches from Cseh et al. [17]. We carry out a comprehensive empirical evaluation on a generated data set that includes both satisfiable and unsatisfiable instances. We analyse the behaviour of our constraint models based on different preference structures, cost functions, and their scalability. We introduce the notion of *important elements*, elaborate on the relation of this notion to relevant notions in the state of the art, and study the behaviour of 3dsm-cyc in the presence of important elements. The results of the evaluation give insight into the convenience of the introduced types of relaxation, in particular in those cases where the four methods agree on the optimal relaxation.

### Structure of the paper

We introduce notation and define the four relaxations of stability formally in Section 2. The constraint models we work on are described in Section 3. Our experimental results are presented in Section 4. A separate section (Section 5) is devoted to the notion of important elements and our experimental results on it. Finally, we conclude in Section 6.

A preliminary version of this study has appeared in the proceedings of CP 2022 [18]. Section 5 contains completely new material compared to that version.

## Notation and problem definitions

In Section 2.1 we formally define input and output formats for 3dsm-cyc, using previous notations [17]. The four ways of relaxing stability are then discussed in Section 2.2. Minimum correction sets are defined in Section 2.3. Finally, matching costs are introduced in Section 2.4.

### Problem definition

#### Input and output

Formally, a 3dsm-cyc instance is defined over three disjoint sets of agents of size *n*, denoted by $A= \{ a_{1}, \dots , a_{n} \}$, $B= \{ b_{1}, \dots , b_{n} \}$, and $C= \{ c_{1}, \dots , c_{n} \}$. A *matching* *M* corresponds to a disjoint set of triples, where each triple, denoted by $(a_i, b_j, c_k)$, contains exactly one agent from each agent set. Each agent is equipped with her own preferences in the input. The cyclic property of the preferences means the following: each agent in *A* has a strict and complete preference list over the agents in *B*, each agent in *B* has a strict and complete preference list over the agents in *C*, and finally, each agent in *C* has a strict and complete preference list over the agents in *A*—where strict and complete list means a strict total order of the set. These preferences are captured by the *rank function*, where $\textrm{rank}_{a_i}(b_j)$ is the position of agent $b_j$ in the preference list of $a_i$, from 1 if $b_j$ is $a_i$’s most preferred agent to *n* if $b_j$ is $a_i$’s least preferred agent.

#### Preferences over triples

The preference relation of an agent on possible triples is derived naturally from the preference list of this agent. Agent $a_i$ is indifferent between triples $(a_i, b_j, c_{k_1})$ and $(a_i, b_j, c_{k_2})$, since she only has preferences over the agents in *B* and the same agent $b_j$ appears in both triples. However, when comparing triples $(a_i, b_{j_1}, c_{k_1})$ and $(a_i, b_{j_2}, c_{k_2})$, where $b_{j_1}\ne b_{j_2}$, $a_i$ prefers the first triple if $\textrm{rank}_{a_i}(b_{j_1}) < \textrm{rank}_{a_i}(b_{j_2})$, and she prefers the second triple otherwise. The preference relation is defined analogously for agents in *B* and *C* as well.

#### Weak and strong stability

A triple $t=(a_i, b_j, c_k)$ is said to be a *strongly blocking triple* to matching *M* if each of $a_i, b_j$, and $c_k$ prefer *t* to their respective triples in *M*. Practically, this means that $a_i, b_j$, and $c_k$ could abandon their triples to form triple *t* on their own, and each of them would be strictly better off in *t* than in *M*. If a matching *M* does not admit any strongly blocking triple, then *M* is called a *weakly stable* matching. Similarly, a triple $t=(a_i, b_j, c_k)$ is called a *weakly blocking triple* if at least two agents in the triple prefer *t* to their triple in *M*, while the third agent does not prefer her triple in *M* to *t*. This means that at least two agents in the triple can improve their situation by switching to *t*, while the third agent does not mind the change. A matching that does not admit any weakly blocking triple is referred to as *strongly stable*. By definition, strongly stable matchings are also weakly stable, but not the other way round. Observe that it is impossible to construct a triple *t* that keeps exactly two agents of a triple equally satisfied, while making the third agent happier, since the earlier two agents need to keep their partners to reach this, which then defines the triple as one already in *M*.

### Relaxing stability

We examine four different ways to relax stability in 3dsm-cyc. Two of them are standard in the stable matching literature and are based on minimising the number of blocking elements, see Section 2.2.1. The other two relaxation notions are introduced in Section 2.2.2, and they build upon elements that are prohibited to be part of a blocking triple. We remark that all four relaxations can be translated to other stable matching problems as well.

#### Almost stable matchings

Let $\textrm{sbt}(M)$ denote the set of strongly blocking triples, and $\textrm{wbt}(M)$ denote the set of weakly blocking triples to a matching *M*. Since strongly blocking triples are also weakly blocking, $\textrm{sbt}(M) \subseteq \textrm{wbt}(M)$.

##### Definition 1

A *strong triple-almost stable* (TAS) matching is a matching that minimises the function $|\textrm{wbt}(M)|$ over all matchings *M*. Analogously, a *weak TAS* matching is a matching that minimises the function $|\textrm{sbt}(M)|$ over all matchings *M*.

If the instance admits a strongly stable matching, then it minimises both functions, but otherwise, there is no connection between the sets of weak TAS and strong TAS matchings.

The agents involved in a strongly blocking triple are called strongly blocking agents, and form the set $\textrm{sba}(M)$. Analogously, agents involved in any weakly blocking triple are called weakly blocking agents, and form the set $\textrm{wba}(M)$. Notice that $\textrm{sba}(M) \subseteq \textrm{wba}(M)$. A natural objective is to find a matching that minimises the functions $|sba(M) |$ or $|\textrm{wba}(M) |$.

##### Definition 2

A matching that minimises $|\textrm{sba}(M) |$ is called *weak agent-almost stable (AAS)*, while a matching that minimises $|\textrm{wba}(M) |$ is called *strong AAS*.

Notice that weak AAS and strong AAS matchings are not identical to weak TAS and strong TAS matchings. As an example, consider two matchings $M_1$ and $M_2$ such that $\textrm{wbt}(M_1)=\{(a_1,b_1,c_1),(a_1,b_1,c_2),(a_1,b_1,c_3)\}$ and $\textrm{wbt}(M_2)=\{(a_1,b_1,c_1),(a_2,b_2,c_2)\}$. We have $|\textrm{wbt}(M_1)|=3$ and $|\textrm{wbt}(M_2)|=2$, so $M_2$ is a better strong TAS candidate than $M_1$. However $|\textrm{wba}(M_1)|=|\{a_1,b_1,c_1,c_2,c_3\}|=5$ and $|\textrm{wba}(M_2)|=|\{a_1,a_2,b_1,b_2,c_1,c_2\}|=6$, so $M_1$ is a better strong AAS candidate than $M_2$.

#### Accommodating elements

Instead of minimising the number of blocking elements, we can eliminate them altogether by setting some agents to be *accommodating*. Accommodating agents never report that they are part of a blocking triple, which eliminates all blocking triples containing at least one of those agents. In a realistic scenario, accommodating agents are allocated compensation for their poor match.

##### Definition 3

A *weak minimally-accommodating stable (MAS)* matching is a matching that minimises the number of accommodating agents needed to eliminate all of its strongly blocking triples. Analogously, a *strong MAS* matching is a matching that minimises the number of accommodating agents needed to eliminate all of its weakly blocking triples.

Notice that MAS matchings are distinct from AAS matchings. As an example, consider the matchings $M_2$ from before, where $\textrm{wbt}(M_2)=\{(a_1,b_1,c_1),(a_2,b_2,c_2)\}$, and the matching $M_3$ such that $\textrm{wbt}(M_3)=\{(a_1,b_1,c_1),(a_1,b_2,c_2),(a_1,b_3,c_3)\}$. We have $|\textrm{wba}(M_2)|=6$ and $|\textrm{wba}(M_3)|=7$, so $M_2$ is a better strong AAS candidate than $M_3$. However, we need both an agent from $\{a_1,b_1,c_1\}$ and an agent from $\{a_2,b_2,c_2\}$ to be accommodating to eliminate the blocking triples in $\textrm{wbt}(M_2)$, while setting a single agent, $a_1$, to be accommodating eliminates all blocking triples in $\textrm{wbt}(M_3)$. Therefore $M_3$ is a better strong MAS candidate than $M_2$.

We can extend the definition of accommodating to groups of agents. Agents *x* and *y* from different agent sets form an *accommodating pair* if they are prevented from appearing *together* in a blocking triple. In 3dsm-cyc, exactly one of the two agents has preferences over the other agent, without loss of generality let us assume that it is *x*. Setting *x* and *y* to be an accommodating pair expresses that *x* receives compensation for not being matched to *y* specifically. However, *x* can appear in a blocking triple with another agent from the set of *y*, and *y* also can block with any other agent than *x*. This compensation is thus less powerful than the previous one.

##### Definition 4

A *weak minimally-pair-accommodating stable (MPAS)* matching is a matching that minimises the number of accommodating pairs needed to eliminate all of its strongly blocking triples. Analogously, a *strong MPAS* matching is a matching that minimises the number of accommodating pairs needed to eliminate all of its weakly blocking triples.

The sets of MPAS and MAS matchings are incomparable. As an example, consider the matching $M_3$ from before, where $\textrm{wbt}(M_3)=\{(a_1,b_1,c_1),(a_1,b_2,c_2),(a_1,b_3,c_3)\}$, and the matching $M_4$ such that $\textrm{wbt}(M_4)={(a_1,b_2,c_3),(a_1,b_2,c_2),(a_2,b_3,c_1)}$. Only the agent $a_1$ needs to be accommodating to eliminate all blocking triples in $\textrm{wbt}(M_3)$, but no single agent appears in all blocking triples of $\textrm{wbt}(M_4)$, so $M_3$ is a better strong MAS candidate than $M_4$. On the other hand, we can eliminate all blocking triples in $\textrm{wbt}(M_4)$ by setting only two pairs to be accommodating, while we need three to do the same for $\textrm{wbt}(M_3)$. Therefore $M_4$ is a better strong MPAS candidate than $M_3$.

Further extending MPAS to groups of three agents would mean minimising the number of accommodating triples, which is equivalent to TAS.

Even though minimising the number of accommodating elements has been first studied in this work, preventing agents or pairs from blocking has been studied in the literature. Instances with pairs that cannot block, but can be part of a matching, have received interest, as they model a ubiquitous scenario in applications [5]. Agents are often not aware of the preferences of others, not even once the matching has been specified. This typically occurs in very large markets, such as job markets [4], or if the preferences are calculated from some data, rather than provided directly by the agents, such as in medical [14] and social markets [3]. Two agents who cannot exchange their preferences form a so-called *free* pair. According to the definition of stability with free pairs, if a matching is only blocked by free pairs, then it counts as stable, as no pair of agents can undermine its stability. The existence of free pairs can only enlarge the set of stable solutions.

Kwanashie [40, Sections 4 and 5] performed an exhaustive study on various stable matching problems with free pairs. Cseh and Heeger [19] extended the notion to preferences with ties and showed further hardness results. The term “stable with free pairs” [14, 19, 28] is equivalent to the adjective “socially stable” [5, 40] for a matching.

In all papers so far, pairs were set to be free at start and the goal was to find an optimal matching with subject to these free pairs. The ability of a pair to block was derived from the instance itself. We emphasise that our approach differs here: we aim to find a strategy to set the fewest elements free to guarantee the existence of a stable solution. The ability to block is therefore not an inherent property of a pair, but can rather be imagined as a switch that can be activated on demand. Besides this, we step into the 3D setting and study free pairs in MPAS and free agents in MAS, both preventing all triples including them from blocking.

Table 1 summarises the four different notions of relaxation that we have explored. We remark that while AAS and TAS require that the relaxation set covers every blocking element, for MAS and MPAS, the relaxation set must hit every blocking element.

**Table 1:** Different ways of interpreting relaxation

	single agent	more than one agent
minimise the number	agent-almost stable	triple-almost stable
of blocking elements	(AAS)	(TAS)
minimise the number of	minimally-accommodating	minimally-pair-accommodating
accommodating elements	stable (MAS)	stable (MPAS)

### Correction sets

The relaxations defined in Section 2.2 give rise to the notion of a correction set.

#### Definition 5

A *correction set* for a matching *M* for AAS and TAS relaxations is a set containing the blocking elements. A *correction set* for *M* for MAS and MPAS relaxations is a set of elements that stabilise *M* if they are set to be accommodating.

Notice that, for all four relaxations, we can have many correction sets for a matching, since any superset of a correction set is also a correction set. A *minimum correction set* for a matching *M* is a correction set for *M* of minimum cardinality. A *minimum correction set* for an instance *I* is a correction set of minimum cardinality across all minimum correction sets for matchings in *I*. While the minimum correction set for a given matching for AAS and TAS relaxations is unique, there might be several minimum correction sets for the same matching for MAS and MPAS relaxations and also several minimum correction sets for an instance for all four relaxations when considering all matchings. An instance admits a stable matching if and only if the size of the minimum correction sets for all relaxation types is 0.

If a matching is blocked by exactly one triple, then the size of its minimum correction set is 1 (for TAS, MAS, and MPAS relaxations) or 3 (for AAS relaxation). If a matching has *b* blocking triples, then the size of its minimum correction set for TAS is *b*, but the minimum correction sets for other relaxation types do not always have the same size. For example, for MAS relaxation, the size can vary from 1 (if the same agent appears in every blocking triple) to *b* (if no two blocking triples contain the same agent).

**Fig. 1:**
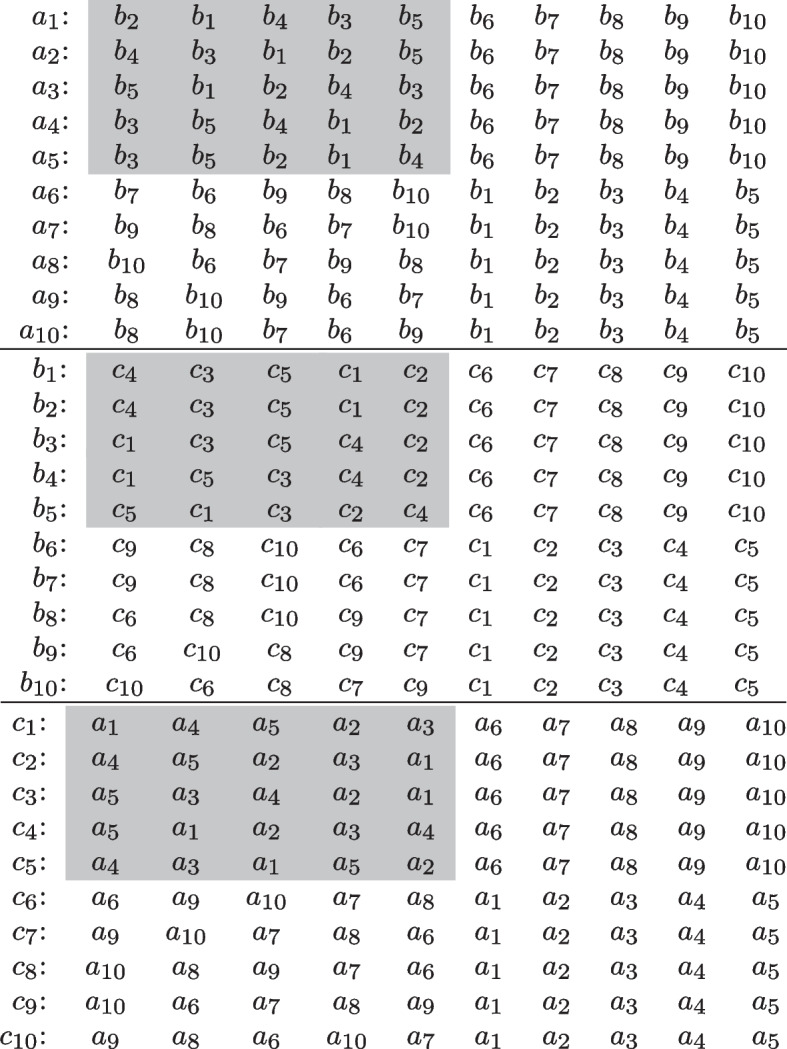
A 3dsm-cyc instance $I_{10}$ built from a 3dsm-cyc instance $I_5$ that does not have a strongly stable matching. The preference lists of the original instance $I_5$ are indicated with a light gray background and correspond to the first half of the preference lists of the first five agents in each agent set of $I_{10}$. The first half of the preference lists of the other five agents in each agent set are renamed copies of the preferences of the agents from $I_5$, so for example if for $1\le i,j,r\le 5$ the agent ranked at position *r* in the preference list of $a_i$ is $b_j$, then the agent ranked at position *r* in the preference list of $a_{i+5}$ will be $b_{j+5}$

The unsatisfiable instances that we experiment on in later sections happen to have a matching with only one blocking triple, meaning that their minimum correction set is a singleton for TAS, MAS, and MPAS relaxations. However, one can design instances with non-singleton minimum correction sets for these relaxation types, for example by copying an instance with a singleton correction set and adding the agents from the other instance to the end of the original preference list. We present such a construction in Fig. 1. In this example, the original instance $I_5$ does not admit a strongly stable matching, meaning that every matching for $I_5$ will have at least one weakly blocking triple. Since $I_{10}$ consists of two copies of $I_5$ glued together as described above, every matching for $I_{10}$ will have at least two disjoint weakly blocking triples, one for each copy of $I_5$, and therefore at least two different agents (respectively pairs) need to be accommodating in order to recover the stability of a matching for MAS (respectively MPAS) relaxation.

We can obtain a minimum correction set of any size by adding more copies of the original unsatisfiable instance. The size of the minimum correction sets for the final instance will be linear in the number *n* of agents in each agent set.

### Matching costs

When computing a minimal set of elements for relaxation, not all agents might be given equal importance. The central authority might allocate a higher compensation to prioritised blocking pairs or to popular agents. For a given relaxation version, the cost of a matching is the sum of the costs of the elements in the minimal set of this particular relaxation. For a given matching *M* and arbitrary costs on agents and triples, we thus have for strong stability:

$$\begin{aligned} \textrm{Cost}_\text {AAS}(M)=&\sum \limits _{a\in \textrm{wba}(M)}\textrm{Cost}(a)\\ \textrm{Cost}_\text {TAS}(M)=&\sum \limits _{t\in \textrm{wbt}(M)}\textrm{Cost}(t)\\ \end{aligned}$$

The definitions for weak stability can be obtained by replacing $\textrm{wbt}$ by $\textrm{sbt}$ and $\textrm{wba}$ by $\textrm{sba}$. For $\textrm{Cost}_\text {MAS}$ and $\textrm{Cost}_\text {MPAS}$, we need a further definition.

#### Definition 6

For a matching *M*, set *S* of agents is *agent-convenient* if setting all agents in *S* to accommodating implies that *M* is stable. Analogously, set *S* of pairs of agents is *pair-convenient* for *M* if setting all pairs in *S* to accommodating implies the stability of *M*.

This definition is the same for both types of stability. We can now write the remaining matching cost definitions for arbitrary agent and pair costs as follows.

$$\begin{aligned} \textrm{Cost}_\text {MAS}(M)=&\underset{S\text { is agent-convenient for }M}{\textrm{min}} \sum \limits _{a\in S}\textrm{Cost}(a)\\ \textrm{Cost}_\text {MPAS}(M)=&\underset{S\text { is pair-convenient for }M}{\textrm{min}} \sum \limits _{p\in S}\textrm{Cost}(p)\\ \end{aligned}$$

Notice that in all four types of relaxation, not specifying element costs is equivalent to having them all set to 1. We will therefore refer to a relaxation as an *arbitrary-cost relaxation* when elements have an explicit cost, and as a *unit-cost relaxation* when they do not.

## Methodology

In this section, we explain how we modified the two best performing models for 3dsm-cyc, called div-ranks and hs [17], to enable them to deal with soft constraints.

### Soft DIV-ranks model

The div-ranks model for 3dsm-cyc with only hard constraints consists of 3*n* variables $X = \{ x_{1}, \dots , x_{n} \}$, $Y = \{ y_{1},\dots , y_{n} \}$, and $Z = \{ z_{1},\dots ,z_{n} \}$, where the domain of each variable *v* is set as $D(v) = \{1,\dots ,n\}$. Assigning $x_{i} = j$ (respectively $y_{i} = j$, or $z_{i} = j$) corresponds to matching $a_{i}$ (respectively $b_{i}$, or $c_{i}$) to her $j^{\text {th}}$ preferred agent. The constraints used to find a stable matching *M*, if any exists, are defined in [17] in the following manner.

- (matching) For all $1\le i,j,k\le n$, the constraint $x_{i}=\textrm{rank}_{a_i}(b_j)\wedge y_{j}=\textrm{rank}_{b_j}(c_k)\Rightarrow z_{k}=\textrm{rank}_{c_k}(a_i)$ is added. This is to ensure that each solution corresponds to a feasible, if not stable, matching. Since domain values correspond to positions in preference lists and not to agents, it is possible for two variables from the same agent set to be assigned the same value. This is why all-different constraints are not used for this model.
- (stability) Under *weak* stability, for all $1\le i,j,k\le n$, the constraint $x_{i}\le \textrm{rank}_{a_i}(b_j)\vee y_{j}\le \textrm{rank}_{b_j}(c_k)\vee z_{k}\le \textrm{rank}_{c_k}(a_i)$ is added. This is to ensure that the triple $(a_i,b_j,c_k)$ is not strongly blocking. When solving the problem under *strong* stability, the inequalities are strict but the following part is added to each disjunction: $\vee (x_{i} = \textrm{rank}_{a_i}(b_j)\wedge y_{j} = \textrm{rank}_{b_j}(c_k)\wedge z_{k} = \textrm{rank}_{c_k}(a_i))$.
- (redundancy) For all $1\le i,j,k\le n$, the constraint $y_{j}=\textrm{rank}_{b_j}(c_k)\wedge z_{k}=\textrm{rank}_{c_k}(a_i)\Rightarrow x_{k}=\textrm{rank}_{a_i}(b_j)$ is added.
- (redundancy) For all $1\le i,j,k\le n$, the constraint $z_{k}=\textrm{rank}_{c_k}(a_i)\wedge x_{i}=\textrm{rank}_{a_i}(b_j)\Rightarrow y_{j}=\textrm{rank}_{b_j}(c_k)$ is added.

For the relaxation version of 3dsm-cyc, we add to the div-ranks model an integer variable $c_{rel}$ corresponding to the cost of the relaxation, as well as additional Boolean variables whose exact number depends on the type of relaxation.

- AAS and MAS: a Boolean variable $relA_i$ for each of the *n* agents $a_i$ in *A*, a Boolean variable $relB_j$ for each of the *n* agents $b_j$ in *B*, and a Boolean variable $relC_k$ for each of the *n* agents $c_k$ in *C*, which amounts to 3*n* additional variables.
- TAS: a Boolean variable $rel_{i,j,k}$ for each of the $n^3$ potential blocking triples $(a_i,b_j,c_k)$.
- MPAS: a Boolean variable $relAB_{i,j}$ for each of the $n^2$ agent pairs $a_i,b_j$ from $A\times B$, a Boolean variable $relBC_{j,k}$ for each of the $n^2$ agent pairs $b_j,c_k$ from $B\times C$, and a Boolean variable $relCA_{k,i}$ for each of the $n^2$ agent pairs $c_k,a_i$ from $C\times A$, which amounts to $3n^2$ additional variables.

For all four types, a variable set to 1 means that its corresponding element is part of the correction set. Determining from the composition of the correction set whether a given triple is allowed to be blocking is expressed in the model by extending the disjunction of the stability constraint corresponding to this triple. The part added depends on the type of the relaxation but not on the kind of stability, so for a given type of relaxation the same part will be added to both weak and strong stability constraints.

- For AAS, we add $\vee (relA_i\wedge relB_j\wedge relC_k)$ to the constraint that checks whether the triple $(a_i,b_j,c_k)$ is blocking. If all three agents are in the correction set, then the constraint is satisfied, and whether this triple is blocking has no effect on the stability of the instance.
- For TAS, we add $\vee rel_{i,j,k}$ to the stability constraint. This immediately satisfies the constraint when the triple is in the correction set.
- For MAS, we add $\vee (relA_i\vee relB_j\vee relC_k)$. Because of the distinction between blocking and accommodating agents, for MAS we only need one agent to be in the correction set for the triple to be disregarded, while for AAS we needed all three agents.
- For MPAS, we add $\vee (relAB_{i,j}\vee relBC_{j,k}\vee relCA_{k,i})$. The constraint is satisfied when any two agents in the triple are present as an accommodating pair in the correction set.

Because relaxation has been added to the stability constraints in a disjunctive way, a trivial solution for the instance can be obtained by assigning 1 to all Boolean variables. Therefore we add a final constraint for the objective function which sums the costs of the elements in the correction set. Minimising this value results in a correction set of minimum cardinality (for unit-cost relaxation), or in a solution of minimum cost (for arbitrary-cost relaxation). Both cases represent a maximum relaxation for the instance. For the unit-cost relaxation, all cost factors in the objective function are replaced by 1.

- For AAS and MAS: $c_{rel}=\sum _{i=1}^n (relA_i\times \textrm{Cost}(a_i))+\sum _{j=1}^n (relB_j\times \textrm{Cost}(b_j))+\sum _{k=1}^n (relC_k\times \textrm{Cost}(c_k))$.
- For TAS: $$c_{rel}=\sum _{i=1}^n\sum _{j=1}^n\sum _{k=1}^n (rel_{i,j,k}\times (\textrm{Cost}(a_i,b_j,c_k)))$$.
- For MPAS: $$c_{rel}=\sum _{i=1}^n\sum _{j=1}^n (relAB_{i,j}\times (\textrm{Cost}(a_i,b_j)))+\sum _{j=1}^n\sum _{k=1}^n (relBC_{j,k}\times (\textrm{Cost}(b_j,c_k)))$$ $$+\sum _{k=1}^n\sum _{i=1}^n (relCA_{k,i}\times (\textrm{Cost}(c_k,a_i)))$$.

### Soft HS model

We extend the hs model from Cseh et al. [17] by relaxing the constraints that enforce the stability of the matching. Following Cseh et al. [17], in the soft hs model, we assume that *T* is the set of all possible triples $$\{(a_1,b_1,c_1), (a_1,b_1,c_2), \ldots , (a_n,b_n,c_n)\}$$, where without loss of generality, the triples in *T* are ordered, that is, $$t_i \in T$$ refers to the $$i^{\text {th}}$$ triple of *T*. We also borrow their definition of non-blocking triples, that is, given a triple $$t \in T$$, we denote by $$ {BT}(t)$$ all the triples in *T* that prevent *t* from becoming a blocking triple given the preferences. The variables and constraints of the model are as follows:

- Let *M* be a set variable whose upper bound is *T*.
- Let *S* be a set variable whose upper bound is as follows.
  - For AAS/MAS: $A \cup B \cup C$
  - For TAS: *T*
  - For MPAS: $$A \times B \cup B \times C \cup C \times A $$
- Let *c* be an integer variable corresponding to the cost of the relaxation.
- (matching) Ensure that each agent from each set is matched by having:
  - $$\forall a \in A : \sum _{t_i \in T : a \in t_i} (t_i \in M) =1$$;
  - $$\forall b \in B : \sum _{t_i \in T : b \in t_i} (t_i \in M) =1$$;
  - $$\forall c \in C : \sum _{t_i \in T : c \in t_i} (t_i \in M) =1$$.
- (stability) In the original version, each stable matching is a hitting set of the non-blocking triples (i.e., $\forall t_j \in T: M \cap \{i: t_i \in {BT}(t_j)\} \ne \emptyset$). We relax this definition as follows.
  - For AAS: $$\forall t_j \in T: \exists \langle a,b,c \rangle \in {BT}(t_j): \langle a,b,c \rangle \in M \vee \{a,b,c\} \subseteq S$$
  - For TAS: $$\forall t_j \in T: \exists t_i \in {BT}(t_j): t_i \in M \vee t_i \in S $$
  - For MAS: $$\forall t_j \in T: \exists \langle a,b,c \rangle \in {BT}(t_j): \langle a,b,c \rangle \in M \vee \{a,b,c\} \cap S \ne \emptyset $$
  - For MPAS: $$\forall t_j \in T: \exists \langle a,b,c \rangle \in {BT}(t_j): \langle a,b,c \rangle \in M \vee \{\langle a,b \rangle , \langle b,c \rangle , \langle c,a \rangle \} \cap S \ne \emptyset $$
- (cost of relaxation) The cost variable is constrained as follows:
  - For AAS/MAS: $c=\sum _{x \in S} Cost(x)$
  - For TAS: $$c=\sum _{\langle a,b,c \rangle \in S} Cost(a,b,c) $$
  - For MPAS: $$c= \sum _{\langle x,y \rangle \in S} Cost(x,y)$$

The type of stability is addressed in the computation of the $$ {BT}$$ sets—the model as such is not concerned with this aspect. In hs, matching *M* is constrained to be a set of triples representing *M* as defined in Section 2.1, so the cost of the relaxation follows the definitions in Section 2.4. In the actual implementation, *M* is represented in terms of an array of $$n^3$$ Boolean variables, where each variable refers to the inclusion/exclusion of the corresponding triple in the mapping. Similarly, *S* is also represented as an array of Boolean variables. The size of this array is either 3*n*, $$3n^2$$ or $$n^3$$, depending on the type of relaxation.

## Experimental results

All experiments were performed on machines with Intel(R) Xeon(R) CPU with 2.40GHz running on Ubuntu 18.04. Tests for the div-ranks model were processed by MiniZinc 2.5.5 [46] before being given to the two constraint solvers Chuffed 0.10.4. [16], which is based on lazy-clause generation, and Gecode 6.3.0 [30]. The hs model on the other hand has been directly encoded using Gecode 6.2.0.

### Dataset

#### Preference lists

The instances used in our experiments belong to three different classes: Random, ML1swap, and ML2swaps. In the latter two, the preferences are based on master lists. Master list instances are instances where the preference lists of all agents in the same agent set are identical. Master lists provide a natural way to represent the fact that in practice agent preferences are often not independent. Examples of their real-life applications occur in resident matching programs [7], dormitory room assignments [51], cooperative download applications such as BitTorrent [2], and 3-sided networking services [22].

The precise method to create an instance from each class is as follows:

- **Random**: generated randomly from uniform distribution.
- **ML1swap**: all agents in the same agent set follow the same randomly chosen master list. Then in each preference list, the positions of two randomly chosen agents are swapped.
- **ML2swaps**: each agent set has a randomly chosen master list that all agents in the set follow. First, two agents are randomly chosen from each agent’s preference list, and their positions are swapped. Then, two more agents from each list are randomly chosen such that the new agents were not involved in the first swap, and their positions are swapped.

For each instance class and each odd size $n\in \{5,7,\dots ,19\}$, we generated instances with *n* agents in each agent set, solved the instances under strong stability, and kept the first 50 that were satisfiable and the first 50 that were unsatisfiable. This gave us a total of 300 instances for each size, 150 with a strongly stable matching and 150 without. We had to restrict ourselves to strong stability for unsatisfiability, because the smallest known instance without a weakly stable matching is of size 90 [41], so it would not have been feasible to obtain a representative sample of reasonably-sized unsatisfiable instances for weak stability.

The three types of instances that we studied have been previously used to test the div-ranks and hs models, along with a fourth class named ML_oneset [17]. Since ML_oneset instances always admit a strongly stable matching [17], we did not include this additional instance class in our experiments.

#### Cost formulas

For each configuration of the model, solver, and relaxation type, each instance was set up with two definitions of costs on its elements. The first one is a unit-cost relaxation, corresponding to a cost of 1 for every agent, pair, and triple in the instance. For the second one, that we call *popularity-cost relaxation*, the cost of an agent is a measure of how well she is ranked in other agents’ preference lists. Formally the cost of an agent $b\in B$ is defined as:

1 $$\begin{aligned} \textrm{Cost}(b)=\sum \limits _{i=1}^n n-\textrm{rank}_{a_i}(b). \end{aligned}$$

The costs of agents from *A* and *C* are defined analogously. The intent is to penalise putting popular agents in the correction set, by giving a higher cost to better ranked agents. The cost of a pair (respectively triple) of agents is the sum of the individual costs of the two (respectively three) agents composing it.

### Scalability

In this section we evaluate the performance of div-ranks and hs by considering how well they scale with respect to the number of agents in the set. We have decided to classify the experiments into eight groups depending on: (a) the satisfiability of the instance, (b) the solver used and (c) whether the soft constraints have unit cost or not.

**Fig. 2:**
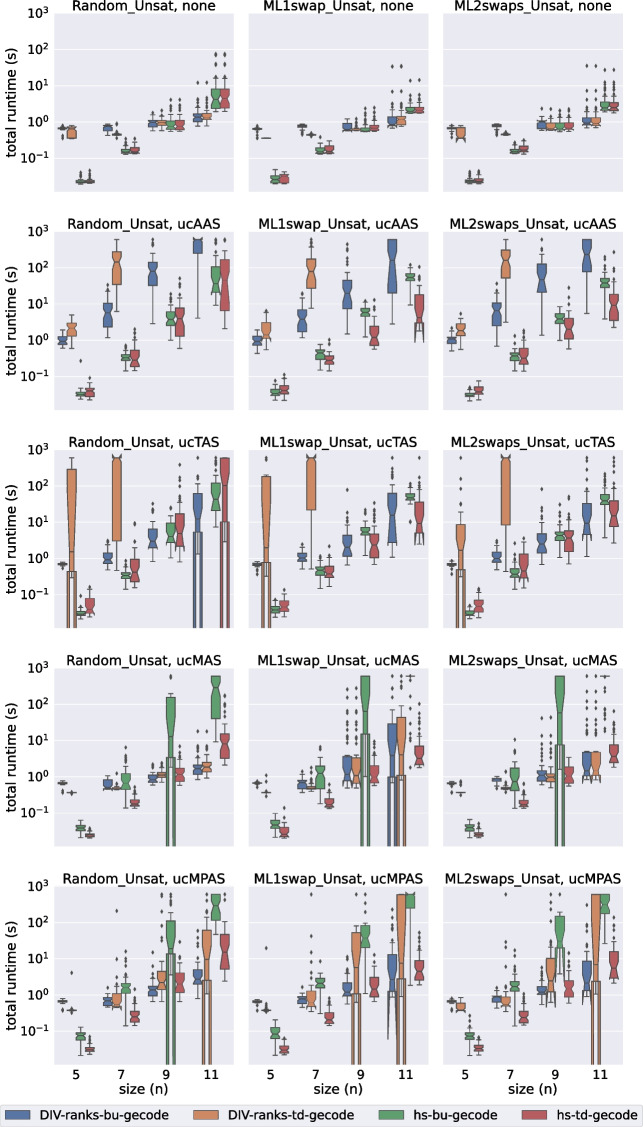
A comparison of total time spent by all Gecode models on the unsatisfiable unit-cost instances

**Fig. 3:**
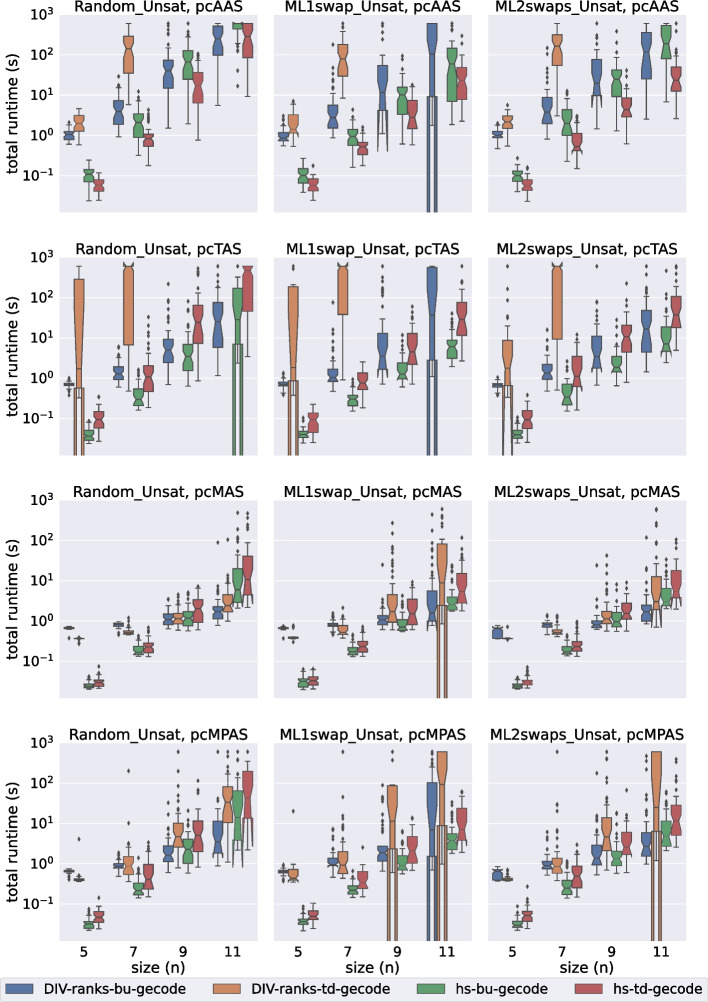
A comparison of total time spent by all Gecode models on the unsatisfiable popularity-cost instances

**Fig. 4:**
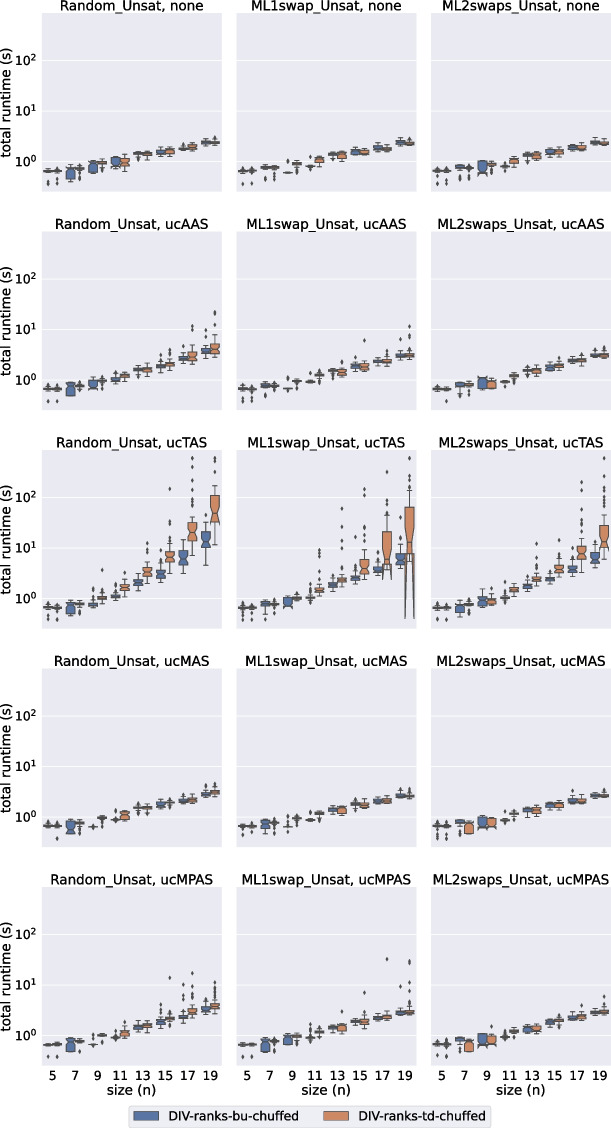
A comparison of total time spent by all Chuffed models on the unsatisfiable unit-cost instances

**Fig. 5:**
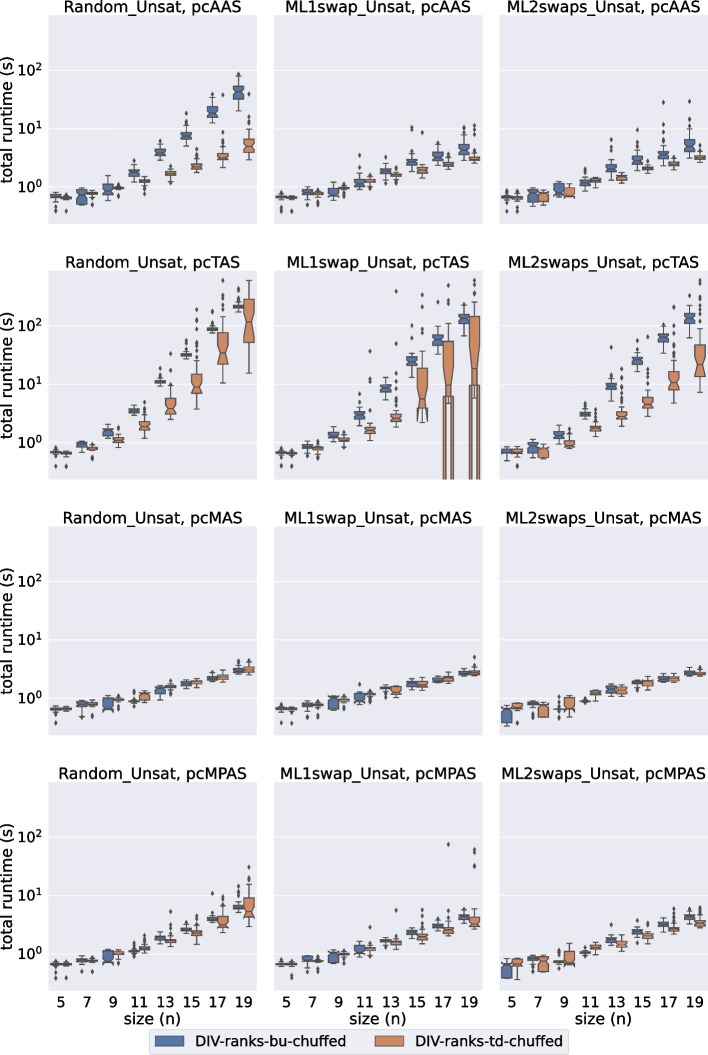
A comparison of total time spent by all Chuffed models on the unsatisfiable popularity-cost instances

The focus of this paper is on dealing with unsatisfiable instances. However, since in practice we cannot always know in advance whether an instance admits a solution, we found it important to check that the satisfiable cases are solved efficiently too. As the instances are satisfiable, the cost of the optimal relaxation is 0 for each one of them, regardless of the type of relaxation. While we could not include the results because of lack of space, all approaches deal with satisfiable instances without major issue.

In Figs. 2, 3, 4, and 5 we present the results for the unsatisfiable instances. The approaches evaluated are classified in terms of: (a) the model used (div-ranks vs hs), (b) the solver used (Gecode vs Chuffed) and (c) the search strategy used (Bottom Up (bu) vs Top Down (td)). *Bottom Up* consists of branching on the cost variable first by selecting the smallest value in the domain first. Effectively this means that we follow a succession of unsatisfiable checks and end with a satisfiable check, which is bound to lead to an optimal solution since we have already proved that there is no solution with a smaller cost. With the *Top Down* strategy we do the opposite: we find a solution and keep on restricting the next one to be better until that is no longer possible. Effectively this means that we follow a succession of satisfiable checks and end with an unsatisfiable check. The unsatisfiable check ensures that the last satisfiable check corresponds to an optimal solution [23]. Our first observation is that the Chuffed approaches clearly outperform the Gecode approaches. As demonstrated by Figs. 4 and 5, all Chuffed approaches solve the vast majority of instances of size 15 in less than 10 seconds, while the Gecode approaches struggle with instances of size 11 in quite a few cases. The Chuffed approaches also result in much fewer failures—in some cases the gap is of more than two orders of magnitude.

We consider 9 relaxation types. The first one (none) corresponds to the case where all soft constraints are considered hard. This category was included to gauge the amount of overhead added by modeling each type of relaxation. The other 8 categories correspond to the unit-cost and popularity-cost versions of the four relaxation options introduced in Section 2.2.

In general we observe that our approaches deal much better with MAS and MPAS than with TAS and AAS. In instances where all relaxation types lead to the same optimal relaxation, we can save a considerable amount of time by computing one of our two relaxation types. When it comes to the type of cost, this does not seem to deteriorate much the performance of the Chuffed approaches. In the Gecode approaches we actually observe an improvement in performance when we consider our popularity-cost instances in most of the cases. The situation might be different for instances with completely arbitrary costs.

The Bottom Up vs Top Down comparison is another point where we observe differences between the Chuffed and the Gecode approaches. In the Chuffed approaches, even though in most of the cases we did not observe major differences, in some cases the Top Down exploration led us to visibly better results. The situation in Gecode is quite the opposite. The very same model (div-ranks) presented very different behaviours depending on whether Top Down or Bottom Up was used. The Bottom Up tests were completed for all the (small) sizes. However, we had to discard some of the Top Down tests since it was already known that they were going to time out. It is important to remark, though, that the Bottom Up strategy did not always lead to improvements. The improvements were mostly observed when dealing with AAS/TAS instances. Similarly, we observed differences in the performance of hs with respect to the Bottom Up vs Top Down comparison. The Bottom Up strategy led us to better results when dealing with the popularity-cost instances in most of the cases.

To see how well our relaxation models scale on larger instances, we chose the best performing approach for each configuration and ran it on unsatisfiable instances with more than 20 agents in each agent set. These instances, 20 in total, were the ones that were determined unsatisfiable for strong stability in the experiments by Cseh et al. [17]. We chose the DIV-ranks model with the Chuffed solver, using the Bottom Up strategy for unit-cost relaxation and Top Down for popularity-cost, because this showed the best performance in our other tests. The results, displayed in Table 2, confirm that it is more efficient to compute MAS relaxations, although AAS and MPAS also scale well for some combinations of instance class and cost function.

**Table 2:** Largest solved instance sizes and smallest unsolved instance sizes for each relaxation version when run with a timeout of one hour, using the DIV-ranks model and the Chuffed solver

Relaxation	Random		ML1swap		ML2swaps	
	largest	smallest	largest	smallest	largest	smallest
	solved	unsolved	solved	unsolved	solved	unsolved
none	35	-	110	-	90	-
unit-cost AAS	35	-	90	110	70	90
popularity-cost AAS	32	35	29	90	70	90
unit-cost TAS	23	29	29	90	35	45
popularity-cost TAS	23	29	29$^\star$	29$^\star$	29	35
unit-cost MAS	35	-	90	110	70	90
popularity-cost MAS	35	-	90	110	70	90
unit-cost MPAS	35	-	90	110	70	90
popularity-cost MPAS	32	35	90	110	70	90

$^\star$: There were two ML1swap instances of size 29 in the dataset. A popularity-cost TAS matching was found before timeout for one but not for the other

## Important elements

### Formal definition

When defining minimum accommodating sets in Section 2.2.2 we assumed that any element can be set accommodating. However, it is a reasonable assumption that certain agents or pairs cannot be compensated to the point of tolerating blocking. We call the set of those elements *important*. With the terminology of free agents or pairs, this means that we restrict the set of elements that can be set free at all.

Formally, an extended version of Definition 3 assumes the existence of a set of important agents $I \subseteq A \cup B \cup C$.

#### Definition 7

The accommodating agents of a *weak minimally-accommodating stable matching with important agents (MASI)* all belong to the complement set of the important agents $(A \cup B \cup C) \setminus I$, and, subject to this, a weak MASI matching minimises the number of accommodating agents needed to eliminate all of its strongly blocking triples. Analogously, a *strong MASI* matching is a matching that minimises the number of accommodating agents needed to eliminate all of its weakly blocking triples, respecting the condition that all accommodating agents belong to the complement set of the important agents.

Definition 4 can be extended analogously. Here our instances have a set of important pairs $I \subseteq (A \times B) \cup (B \times C) \cup (C \times A )$ instead of agents. The goal is to find a matching that can be stabilised by setting as few pairs, all outside of this set, accommodating as possible.

#### Definition 8

In a *weak minimally-pair-accommodating stable (MPASI) matching with important pairs*, only pairs in the complement set of the important pairs $(A \times B) \cup (B \times C) \cup (C \times A ) \setminus I$ are set to be accommodating. Subject to this, a weak MPASI matching minimises the number of accommodating pairs needed to eliminate all of its strongly blocking triples. Analogously, a *strong MPASI* matching is a matching that minimises the number of accommodating pairs needed to eliminate all of its weakly blocking triples, respecting the condition that all accommodating pairs belong to the complement set of the important pairs.

Relaxation without important elements is a pure optimisation problem, because a solution can always be found by setting all relaxation variables to 1, or, in other words, by setting all triples free and thus stopping them from blocking. Adding important elements restricts the set of blocking triples that can be ignored, making the question of satisfiability relevant again.

To implement important elements in our model, we set the relaxation variable corresponding to each one to false. For example, if agent $a_i$ is important, then variable $relA_i$ in the div-ranks model of MAS is set to 0. If pair $(b_j,c_k)$ is important, then variable $relBC_{j,k}$ in the div-ranks model of MPAS is set to 0.

### Experimental results

We now experimentally study the behavior of the problem in the presence of important elements. In particular, we seek to answer two questions:

1. How many important elements can we add without increasing the minimum number of accommodating elements needed to find a solution?
2. What is the impact of important elements on the runtime?

We tested instances for both MAS and MPAS relaxation types. Since we were interested in how important elements change the size of the minimum correction set, we did not assign an individual cost to each element, meaning that we are solving unit-cost relaxation. To run the experiments, we chose the approach with the best performance for this kind of instances: the Chuffed solver on the div-ranks model with Bottom Up strategy. We used the same hardware as in Section 4.

#### Dataset

We based our dataset on unsatisfiable instances with *n* agents in each agent set, for each $n\in \{5,8,11,14,17,20\}$. For each size *n*, the composition of the dataset is:

- 50 Random instances;
- 50 ML_1swap instances;
- 50 ML_2swaps instances.

All instances were generated with the same method and seed as the instances in Section 4, so when the sizes coincide, the instances are the same. We also experimented on ten instances with $23\le n\le 29$, all picked from Section 4’s dataset of large instances.

For each instance and each relaxation type, we generated the following sets of important elements.

- 1 empty set, to serve as reference.
- 20 singleton sets. For MAS, the agent is randomly chosen among all agents in the instance. For MPAS, the first agent of the pair is the one in the MAS set, and the agent ranked first by this agent is then picked to complete the pair. For example, if the first of the 20 sets in the MAS dataset consists of the agent $c_k$, then the first of the 20 sets in the MPAS dataset will consist of the agent $c_k$ and the agent from *A* ranked first in the preference list of $c_k$. The reason for not simply picking a random pair for the MPAS set is twofold:
  - Because of the cyclic nature of the problem, setting a single pair to be important will affect one of its two agents far more than the other.
  - The idea behind setting an element important is to protect its preferences, and it does not make much sense to protect a random preference without protecting the one ranked first.
- 20 sets containing a number of elements equal to 90% of the total number of elements, rounded down. Recall that for MAS the total number of elements is 3*n*, while for MPAS it is $3n^2$. The elements in these sets are chosen completely at random, for both MAS and MPAS.

All sets are guaranteed to be distinct. For $n=5$, the number of sets in each category is only 15, because instances of each size only contain 15 agents, so we cannot have more than 15 distinct singleton sets for MAS.

**Fig. 6:**
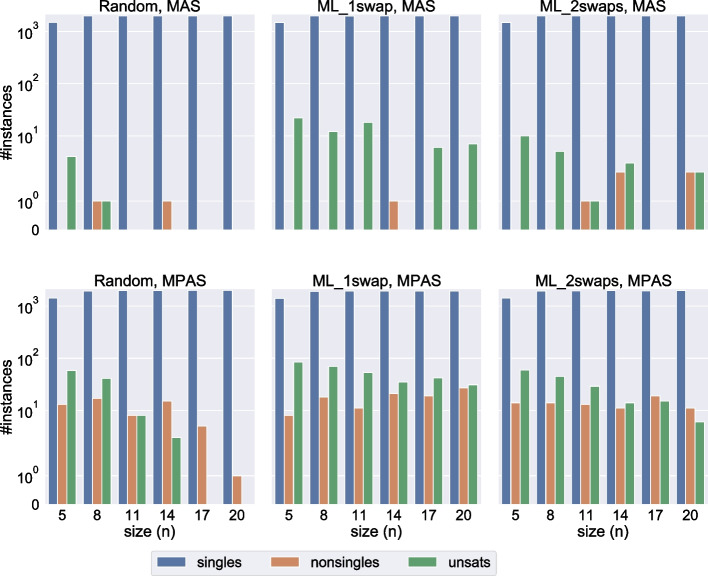
Number of cases with a singleton/non-singleton/no correction set when the number of important elements is either one, or 90% of the total number of elements

**Fig. 7:**
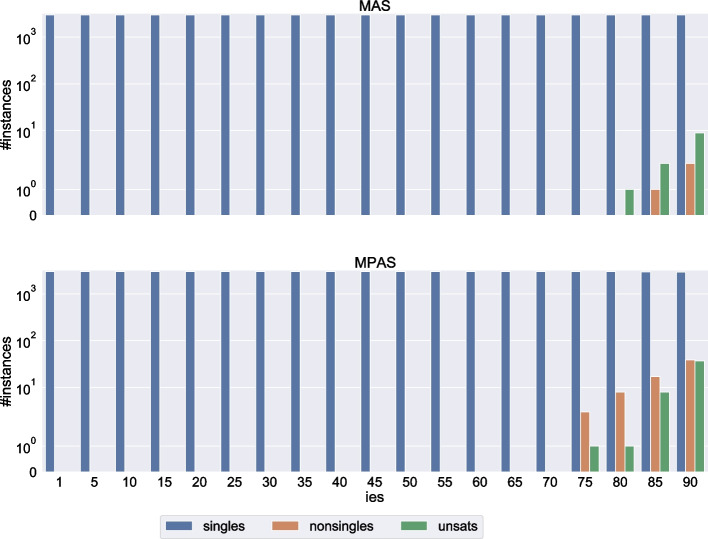
Number of cases with a singleton/non-singleton/no correction set for size 20 instances. *ies* is the percentage of elements that are important, except for $ies=1$ which designates singleton sets

Testing singleton sets and sets containing almost all elements allows us to study both extremes. To get a more refined view, we generated additional sets for sizes $n\ge 20$. More precisely, for every $p=5,10,15,\dots ,80,85$ we also generated 20 sets containing a number of elements equal to $p\%$ of the total number of elements, rounded down. As it was the case with the 90% sets, all elements are chosen completely at random for both MAS and MPAS and all sets are guaranteed to be distinct.

We tested our model on each combination of instance, relaxation type and set of important elements. This amounted to 62 tests for each instance of size $n=5$; 82 tests for each instance of size $5<n<20$; and 762 tests for each instance of size $n\ge 20$, for a total of 180,420 tests.

#### Impact on the correction set

In all the experiments with no important elements we observed that the correction sets were singleton, i.e., it was enough to set one element accommodating to regain stability. But as the number of important elements increases, the pool of available elements for the correction set shrinks, and when all the elements that could be accommodating by themselves become important, there are no longer singleton correction sets. Sometimes the only impact is that the minimum correction set is larger with important elements than without. Other times, even placing all elements that are not important in the correction set is not enough to regain stability, and the instance becomes unsatisfiable.

**Fig. 8:**
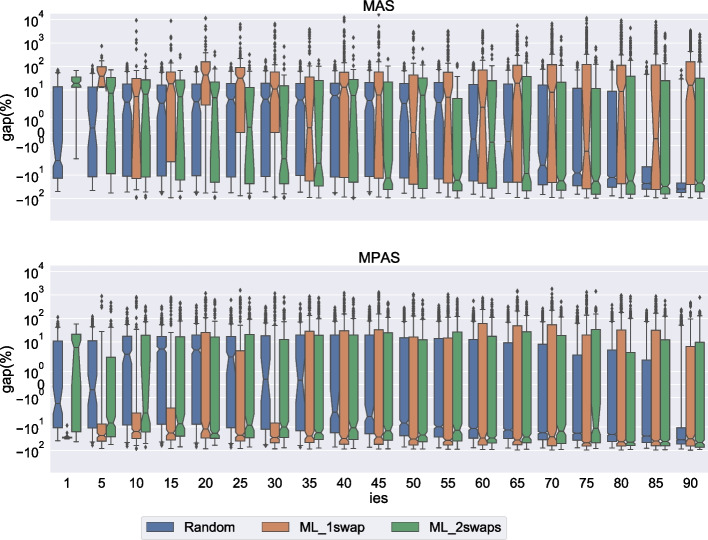
Impact of important elements on the runtime for size 20. The plot shows the gap wrt to the time taken when no important elements are present

**Fig. 9:**
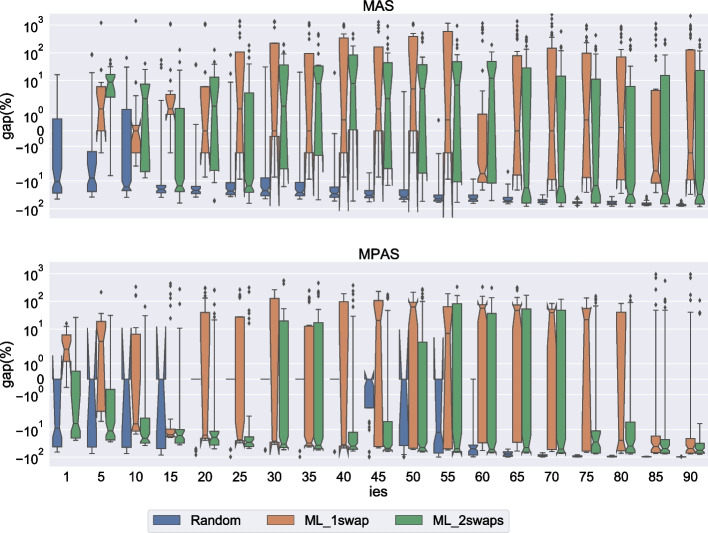
Impact of important elements on the runtime for size 29. The plot shows the gap wrt to the time taken when no important elements are present

We report the different types of correction sets depending on instance size in Fig. 6. We see that, even with important elements, the correction sets remain singleton in the vast majority of cases. The number of other cases seems to remain constant, or even slightly decreases, as the instance size increases.

In Fig. 6 we only reported results for two types of sets of important elements: singletons and sets that contain almost all elements. Of course, the impact of important elements on the correction set will increase with their number; a higher number of important elements means a higher likelihood of either the minimum correction set containing more than one accommodating element, or of the instance being unsatisfiable. We show the exact threshold at which these events happen for size 20 instances in Fig. 7. In the figure, all three generation methods are combined, so for each combination of the relaxation type (MAS or MPAS) and number *p* of important elements, we have 3,000 cases (150 instances times 20 sets of *p* important elements). As we empirically show with these results, the minimum correction set will still be a singleton even when most of the agents are important, and we can answer the first question asked at the beginning of Section 5.2:

1. How many important elements can we add without increasing the minimum number of accommodating elements needed to find a solution?

- 75% of all elements for MAS relaxation.
- 70% of all elements for MPAS relaxation.

#### Impact on runtime

We also measure the impact important elements have on the runtime taking as point of reference the runtime observed when no important elements are present. That is, for a given instance, the gap reported in Figs. 8 and 9 is defined as $$\frac{t'-t}{t}\cdot 100$$, where *t* is the runtime when no important elements are considered and $$t'$$ is the runtime when the important elements are considered. In Figs. 8 and 9 we focus on the second set of instances described in Section 5.2.1. In particular, we report the results of sizes 20 and 29 since for these sizes we have data for all generation types (i.e., Random, ML_1swap and ML_2swaps). Remember that for this second set of experiments we generated, for every $$p=5,10,15,\dots ,80,85,90$$, twenty sets containing a number of elements equal to $$p\%$$ of the total number of elements, rounded down (in addition to the singleton sets generated). In the figures, for each *p* we report the gap considering the relaxation type and the generation type. By $$ {ies}=1$$ we mean the instances associated with singleton important element sets. The other values of $$ {ies}$$ correspond to the other values of *p*.

For both $n=20$ and $n=29$ the gap is mostly negative. That is, the problem often gets easier with the presence of important elements. The decrease in runtime is more noticeable for random instances with a lot of important elements in $n=29$ (see Fig. 9) where we observe a consistent gap of almost $100\%$. For this size, we also observe a clear separation between the random instances and the non-random instances.

In a few cases, most noticeably for size 29 ML_1swap instances under MPAS relaxation with a number of important elements between 45% and 75% of the total number of elements, we observe a significant positive gap indicating that in those cases the problem gets significantly harder with the presence of important elements. We recall that in these experiments we are considering a timeout of one hour, which prevents us from observing more significant positive gaps associated with high runtimes. The fact that most of these positive gaps can be found when the number of important elements is neither very large nor very small, hints to the presence of a peak of difficulty.

We can use our empirical observations to answer the second question asked at the beginning of Section 5.2:

2. What is the impact of important elements on the runtime?

- Important elements make the problem easier when they are either few or plentiful.
- Important elements can sometimes make the problem harder if their number is within a certain range, depending on generation method and relaxation type.

## Conclusion and future work

We extended 3dsm-cyc constraint models to four relaxation versions of the problem, two based on already established two-dimensional relaxation notions, and two that we introduced. For each of these four relaxations, we tested our models on instances of various sizes and types, for two different cost functions, and using both a bottom-up and a top-down approach. Our results show that our models are able to efficiently compute a maximum relaxation for unsatisfiable 3dsm-cyc instances.

We introduced the notion of *important elements*, elaborated on the relation of this notion to relevant notions in the state of the art, and studied the behaviour of 3dsm-cyc in the presence of important elements. We concluded that a large number of important elements can be added without increasing the minimum number of accommodating elements needed to find a solution. We also observed that important elements make the problem easier when they are either few or plentiful, and in some cases make the problem harder when the number of important elements is within a certain range, depending on the generation method and relaxation type.

While our relaxation models performed well for the two cost functions that we studied, it would be interesting to know in what ways their behavior would be affected when given different formulas for the costs of the elements in the instance. For example, one could set the cost of a triple as the difference between the highest and lowest costs of its agents, mirroring the definition of sex-equal [37, 45, 56, 60] optimisation for satisfiable instances.

Another possible avenue of research would be to explore the relations between minimum correction sets of different relaxation types. If for a particular class of instances the maximum relaxations are identical for different types, then one could use our findings that the two new relaxation versions lead to better performance, and search for minimally-accommodating stable matchings instead of almost stable matchings to get the same result faster.
